# Tuning of the Structure and Magnetocaloric Effect of Mn_1−x_Zr_x_CoGe Alloys (Where x = 0.03, 0.05, 0.07, and 0.1)

**DOI:** 10.3390/ma14113129

**Published:** 2021-06-07

**Authors:** Karolina Kutynia, Piotr Gębara

**Affiliations:** Department of Physics, Częstochowa University of Technology, Armii Krajowej 19, 42-200 Częstochowa, Poland; karolina.kutynia@pcz.pl

**Keywords:** magnetocaloric effect, Heusler alloys, X-ray diffraction

## Abstract

The aim of the present work is to study the influence of a partial substitution of Mn by Zr in MnCoGe alloys. The X-ray diffraction (XRD) studies revealed a coexistence of the orthorhombic TiNiSi-type and hexagonal Ni_2_In- type phases. The Rietveld analysis showed that the changes in lattice constants and content of recognized phases depended on the Zr addition. The occurrence of structural transformation was detected. This transformation was confirmed by analysis of the temperature dependence of exponent n given in the relation ΔS_M_ = C·(B_MAX_)^n^. A decrease of the Curie temperature with an increase of the Zr content in the alloy composition was detected. The magnetic entropy changes were 6.93, 13.42, 3.96, and 2.94 J/(kg K) for Mn_0.97_Zr_0.03_CoGe, Mn_0.95_Zr_0.05_CoGe, Mn_0.93_Zr_0.07_CoGe, and Mn_0.9_Zr_0.1_CoGe, respectively. A significant rise in the magnetic entropy change for samples doped by Zr (x = 0.05) was caused by structural transformation.

## 1. Introduction

The magnetocaloric effect (MCE) is the change of temperature of magnetic material under the variations of an external magnetic field. This effect is manifested as the cooling or heating of magnetic materials under the influence of an alternating magnetic field [1]. The MCE is observed in all magnetic materials. It is the result of the coupling of the magnetic field with the magnetic subnetwork, which leads to a change in the magnetic part of the entropy of the solid [2]. This phenomenon is described as the adiabatic temperature change (∆T_ad_) or magnetic entropy change (∆S_M_).

The most popular magnetocaloric materials include pure Gd and its alloys [3], La(Fe, Si)_13_ alloys [4,5], and manganites [6]. The magnetocaloric effect is observed in a group of alloys called Heusler alloys. This is a group of chemical compounds and alloys. Recently, research has focused on full Heusler [7] and half Heusler [8] alloys, such as: (MnNiGe)_1−x_-(FeCoGe)_x_ [9], Co_1−x_Cu_x_MnSb [10], NiFeSb [11], CoV_1−x_MnSb, NiTi_1−x_Mn_x_Sb [12], Co(Mn, Nb)Sb [13], (Zr_0.5_Hf_0.5_)Co(Sb_0.85_Sn_0.15_) [14], MnFeP_1−x_Ax_x_ [15], and MnCoGe [16]. 

**Figure 1:**
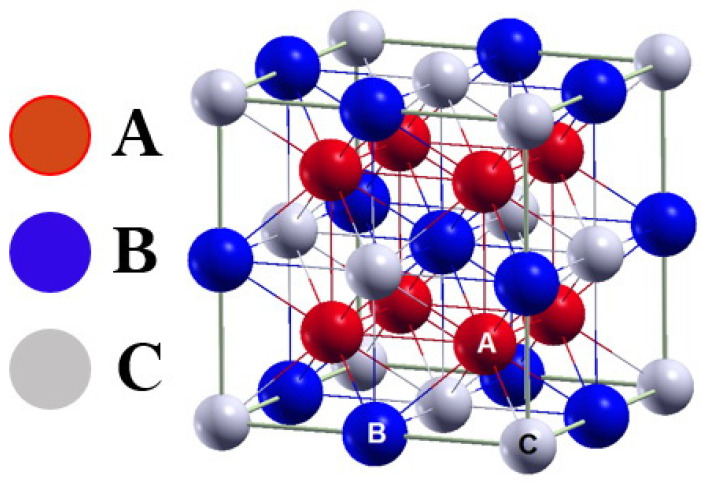
Full Heusler alloy structure model [19].

The general formula for describing full Heusler alloys is: X_2_YZ, where X and Y are atoms from the subgroup (transition metal), and Z is atoms from the main group (metalloids) [17]. The characteristic feature of the full Heusler is: 2: 1: 1 stoichiometry, the structure of the Cu_2_MnAl type, as well as the Fm$\bar{3}$m space group (No. 225, L2_1_). The structure of L2_1_ consists of four interpenetrating cubic subnets with a face-centered (fcc) [18]. The atoms are in the following positions: A—4a (0, 0, 0), B—4b (0.5, 0.5, 0.5), and C—8c (0.25, 0.25, 0.25). Metals are most often included among these alloys [19]. The structure of a full Heusler is shown in Figure 1.

**Figure 2:**
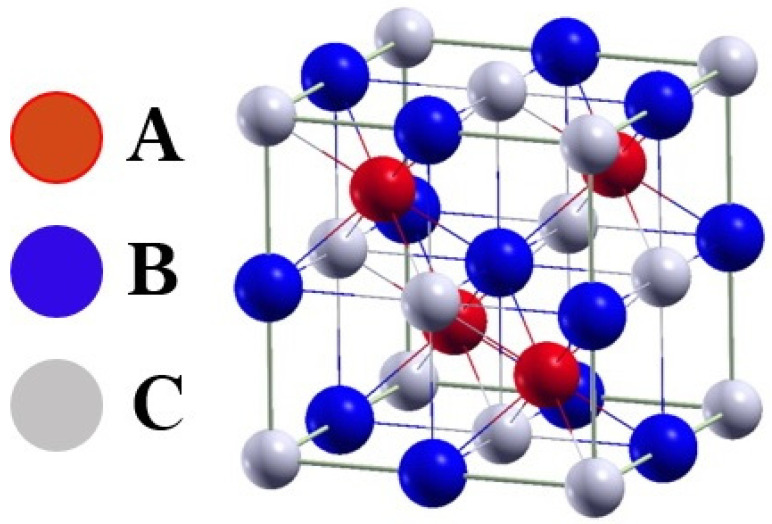
Half-Heusler alloy structure model [20].

The general formula for half-Heusler alloys is: XYZ. Characteristic features of this type of alloy include 1:1:1 stoichiometry, structure of the MgAgAs type, as well as the F$\bar{4}$3m space group (No. 216, C1_b_). The structure of C1_b_ is obtained by removing one location of the X atom from the L2_1_ structure [18]. The half-Heusler alloy atoms are in the following positions: A—4a (0, 0, 0), B—4b (0.5, 0.5, 0.5), and C—4c (0.25, 0.25, 0.25). These alloys include most often semiconductors [20]. The half-Heusler structure is shown in Figure 2.

The influence of partial substitution of Mn by Zr was previously studied in [21,22]. Qian and coworkers showed the possibility of the induction of martensitic transition for the specific composition of the (Mn,Zr)CoGe alloy. However, they did not present complete analysis of the order of phase transition or i.e., values of the refrigeration capacity. In order to broaden the knowledge concerning on this group of materials, we decided to study them. The aim of the present study was to investigate the effect of the substitution of Mn by Zr in the MnCoGe alloy on the structure, magnetic properties, and phase transition.

## 2. Sample Preparation and Experimental Details

Samples with the nominal composition of Mn_1−x_Zr_x_CoGe, where x = 0.03, 0.05, 0.07, and 0.1, were prepared using the arc melting method of high purity elements in an Ar protective gas atmosphere. The samples were remelted several times in order to ensure their homogeneity. The X-ray diffraction (XRD) studies were performed using a Bruker D8 Advance diffractometer (Bruker, Karlruhe, Germany) with CuKα radiation and a LynxEye semiconductor detector (Bruker, Karlruhe, Germany). The collected X-ray pattern was analyzed with the Bruker EVA software (4.3). The Rietveld analysis was conducted using the PowderCell 2.4 package [23]. Magnetic measurements were carried out using the Quantum Design Physical Properties Measuring System (PPMS) model 6000, equipped to work with a wide range of magnetic fields and temperatures.

## 3. Results and Discussion

**Figure 3:**
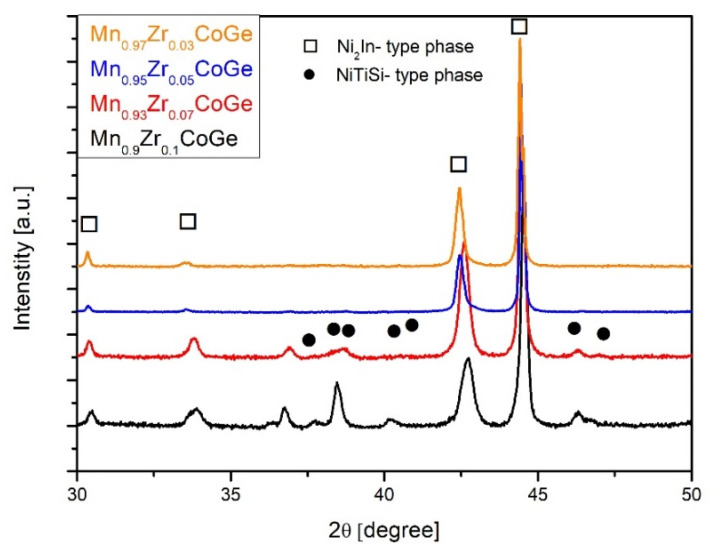
The XRD patterns collected for all studied samples.

The room temperature XRD patterns were measured for all investigated samples and are presented in Figure 3. For the Mn_0.97_Zr_0.03_CoGe alloy sample, the dominant hexagonal Ni_2_In- type phase was detected with small amount of the orthorhombic NiTiSi-type phase. A similar phase composition was observed for samples of the Mn_0.95_Zr_0.05_CoGe alloy. An intensive growth of the orthorhombic NiTiSi-type phase, at the expense of the Ni_2_In- type phase, was observed for samples doped with Zr for x = 0.07 and x = 0.1. Qian and coworkers in [22] showed that the partial substitution of Mn by Zr caused lowering of the temperature of structural transition and induced the formation of the hexagonal Ni_2_In-type phase. 

However, current studies confirmed the results described in [21]. A slight increase of lattice constants is visible with an increase of the Zr content in the alloy composition. Such an effect was expected due to the ionic radius of Zr (r_Zr_ = 1.60 Å) being higher than Mn (r_Zr_ = 1.18 Å), and this causes expansion of the orthorhombic and hexagonal phases. Considering that the orthorhombic structure could be considered as a distorted hexagonal cell [24], an addition of Zr promotes the formation of the NiTiSi-type phase. Deep analysis of the XRD patterns did not detect any additional phase related to an occurrence of impurities. The Rietveld analysis carried out using experimental XRD patterns revealed some slight changes in the lattice constants of recognized phases. The results of the Rietveld refinement were collected in Table 1.

Johnson in [25] showed relations between the unit cells of these two structures as:

(1) $$\begin{array}{l} a_{orth}=c_{hex}\\ b_{orth}=a_{hex}\\ c_{orth}=\sqrt{3}a_{hex} \end{array}$$

**Figure 4:**
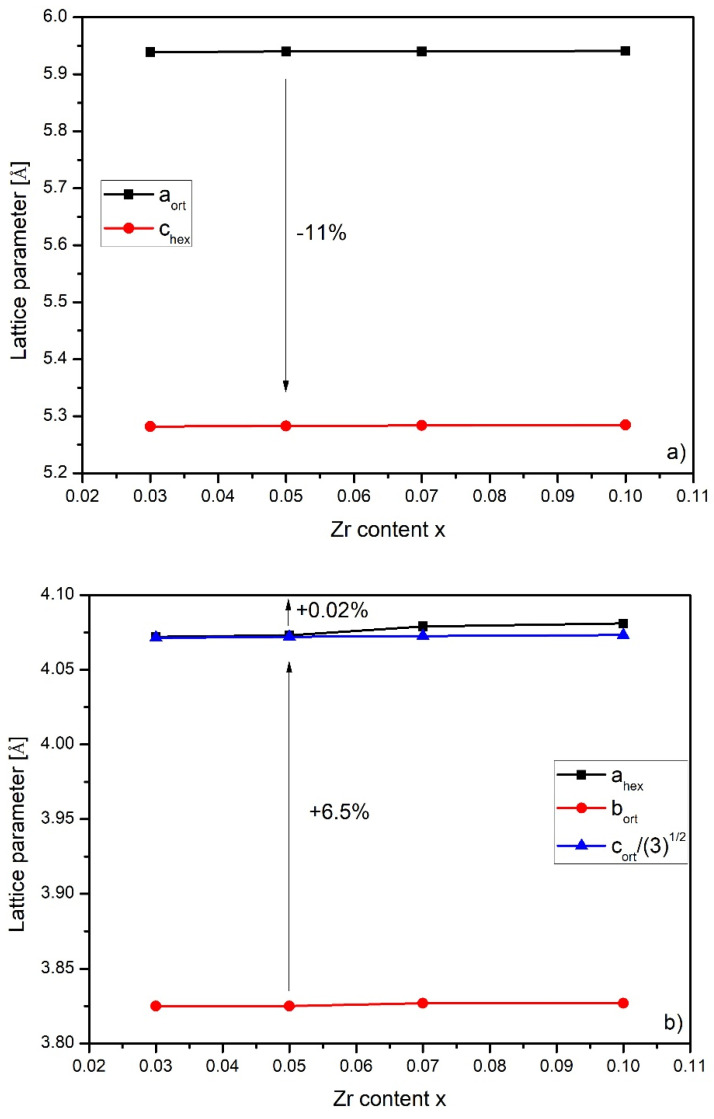
The Zr content dependence of the lattice constant of the analyzed unit cells. Errors were not matched as they were smaller than the symbol size. (**a**) Zr content of lattice parameters (a_ort_ and c_hex_); (**b**) Zr content of lattice parameters (a_hex_, b_ort_ and c_ort_/(3)^1/2^).

Figure 4 presents the dependence between lattice parameters versus the Zr content of the hexagonal and orthorhombic structure for the studied samples. It is clearly seen that the orthorhombic phase changed by 11% along the a axis during the orthorhombic-hexagonal structural transition. At the same time, this lattice enlarged by 6.5% and 0.02% along the b and c axes, respectively. An occurrence of structural transformation significantly affected the value of the magnetic entropy change. The obtained values correspond well with the results delivered in [22,26]. As it was shown by Gschneidner and coworkers in [27], such a structural transformation could increase ΔS_M_ by even 90%.

**Figure 5:**
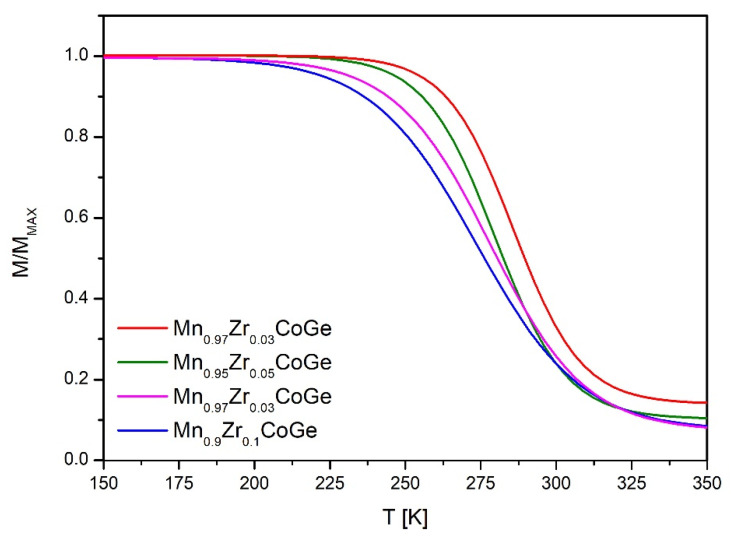
The temperature dependences of magnetization collected under the external magnetic field of 0.01 T for all studied samples. Values were normalized to their maximum value.

To measure the Curie point, the temperature dependences of magnetization were collected in a magnetic field of 0.01 T for all studied samples (Figure 5). The Curie temperature was revealed by calculations of the first derivative of the M = f(T) curves. The estimated values of the T_C_ were 290, 285, 283, and 278 K for Mn_0.97_Zr_0.03_CoGe, Mn_0.95_Zr_0.05_CoGe, Mn_0.93_Zr_0.07_CoGe, and Mn_0.9_Zr_0.1_CoGe, respectively. The gradual decrease of the T_C_ was observed, which is expected in accordance with previous studies [15,21]. Such behavior could also be caused by lowering of the magnetic moment of Mn by Zr during mixing as was shown in [28].

The magnetocaloric effect was studied indirectly by calculations of the magnetic entropy change ΔS_M_. In order to calculate the ΔS_M_ values, the magnetic isotherms were measured for a wide range of temperatures. The calculations of magnetic entropy change were realized using the Maxwell relation [29]:

(2) $$\Delta S_{M}(T,\Delta H)=\mu_{0}\int_{0}^{H}{\left(\frac{\partial M(T,H)}{\partial T}\right)}_{H}dH$$

where μ_0_ is the magnetic permeability, H is the magnetic field strength, M is the magnetization, and T is the temperature.

Equation (2) was implemented in Mathematica software using the following algorithm:

(3) $$\Delta S_{M}(\frac{T_{i}+T_{i+1}}{2})\approx \frac{1}{T_{i+1}-T_{i}}\left[\int_{0}^{B_{\max}}M(T_{i+1},B)dB-\int_{0}^{B_{\max}}M(T_{i},B)dB\right]$$

where B is the magnetic field induction according to the relation B = μ_0_H.

**Figure 6:**
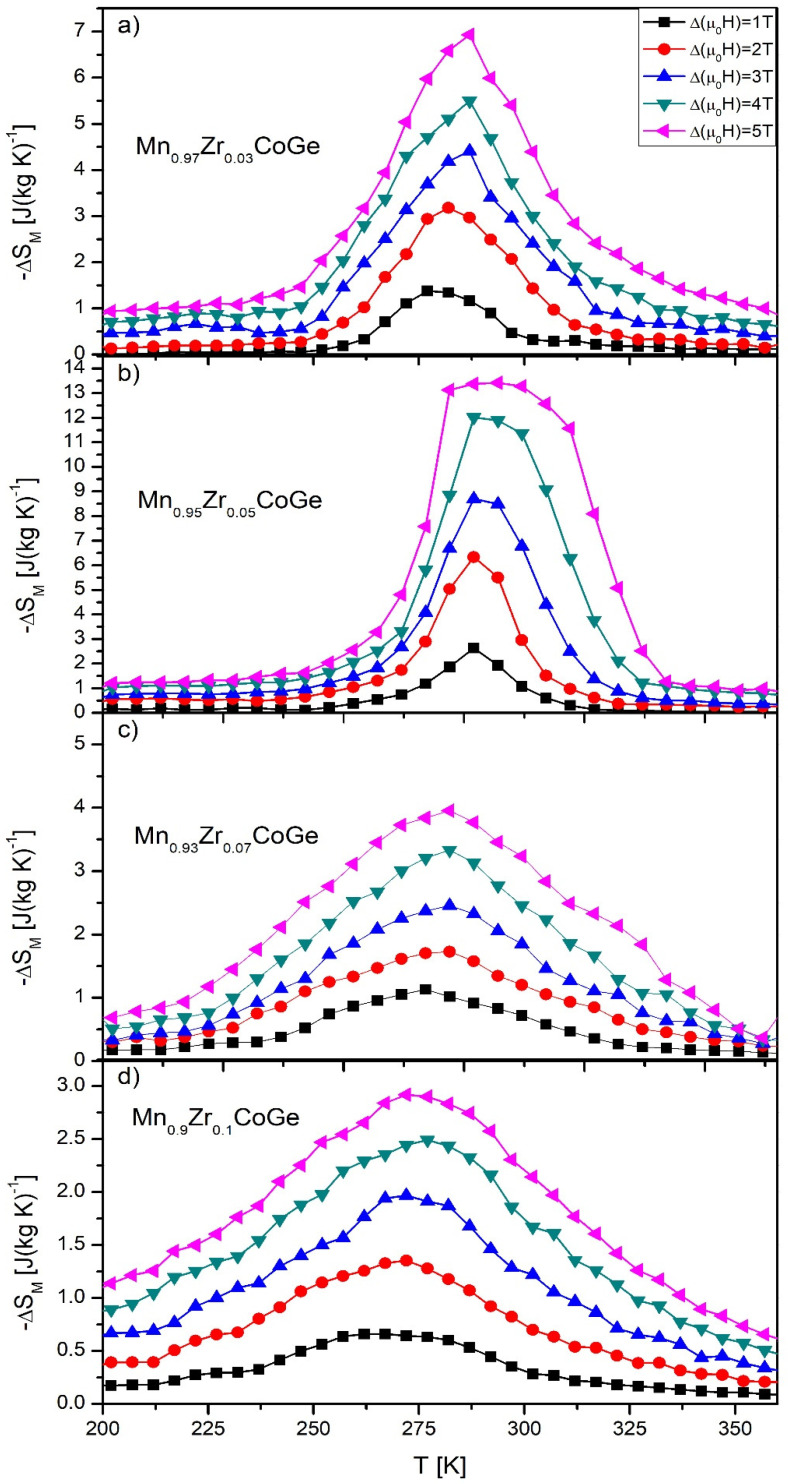
The temperature dependences of the magnetic entropy changes calculated for Mn_0.97_Zr_0.03_CoGe (**a**), Mn_0.95_Zr_0.05_CoGe (**b**), Mn_0.93_Zr_0.07_CoGe (**c**), and Mn_0.9_Zr_0.1_CoGe (**d**) alloys.

**Figure 7:**
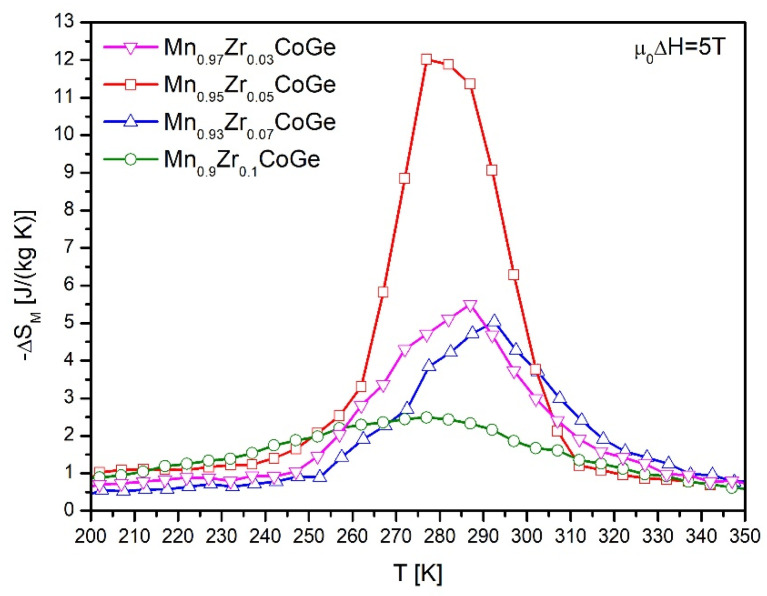
The temperature dependences of the magnetic entropy changes calculated for Mn_0.97_Zr_0.03_CoGe (**a**), Mn_0.95_Zr_0.05_CoGe (**b**), Mn_0.93_Zr_0.07_CoGe (**c**), and Mn_0.9_Zr_0.1_CoGe (**d**) alloys under the change of external magnetic field ~5 T.

The temperature evolution of the magnetic entropy change is presented in Figure 6. The highest values of the ΔS_M_ calculated for the change of external magnetic field ~5 T, were 6.93, 13.42, 3.96, and 2.94 J/(kg K) for Mn_0.97_Zr_0.03_CoGe, Mn_0.95_Zr_0.05_CoGe, Mn_0.93_Zr_0.07_CoGe, and Mn_0.9_Zr_0.1_CoGe, respectively (Figure 7). In comparison to the results reported for the MnCoGe-based alloy presented in [21], the Zr addition caused a slight decrease of the ΔS_M_. However, a further increase to x = 0.05 induced a significant rise of the magnetic entropy change. Moreover, the asymmetric shape of the ΔS_M_ vs. T curve for the Mn_0.95_Zr_0.05_CoGe alloy sample suggests that such a relevant increase was caused by a magnetostructural first order phase transition. 

Further increases of the Zr content in the alloy composition caused a decrease of the ΔS_M_. The values of magnetic entropy change are comparable with those reported in [26] However, these values are almost two times lower than results presented by Qian et al. [22]. Moreover, for samples with the highest Zr content (x = 0.07 and 0.1), a significant broadening of the ΔS_M_ peak was noticed.

In order to conduct more deep characterization of the magnetocaloric properties, the refrigeration capacity was calculated using the following relation [30]:

(4) $$\mathrm{RC}(\delta T,H_{\mathrm{MAX}})=\int_{T_{\mathrm{cold}}}^{{}_{T_{\mathrm{hot}}}}\Delta S_{M}(T,H_{\mathrm{MAX}})dT$$

where RC is the refrigerant capacity, δT = T_hot_ − T_cold_ is the temperature range of the thermodynamic cycle (δT corresponds to the full width at half maximum of magnetic entropy change peak), and H_MAX_ is the maximum value of the external magnetic field. 

The highest value of the RC was reached for the sample with the Zr content x = 0.05. This was caused by the relatively high value of the magnetic entropy change. In the case of other studied alloys, the RCs were similar. The values of magnetic entropy change ΔS_M_ and refrigeration capacity RC are collected in Table 2.

As it was mentioned above, the significant rise of magnetic entropy change could be related to the first order phase transition. An interesting and relatively fast technique to investigate the order of phase transition was proposed by Law et al., which could be called the Law–Franco method [31]. This technique is based on the phenomenological field dependence of magnetic entropy change proposed by Franco in [32] and described by the following relation:

(5) $$\Delta S_{M}=C\cdot {\left(B_{\mathrm{MAX}}\right)}^{n}$$

where C is a constant depending on temperature and n is the exponent related to the magnetic state of sample. The n exponent can be easily calculated by modification of Equation (5) in the form proposed in [33]:

(6) $$\ln\Delta S_{M}=\ln C+n\ln B_{\mathrm{MAX}}$$

The n exponent is strongly dependent on the magnetic state [34]. If a material obeys the Curie law, the exponent n = 1 in the ferromagnetic state (below T_C_), and n = 2 in the paramagnetic state (above T_C_). The exponent n value at the Curie point is described by the relation:

(7) $$n=1+\frac{1}{\delta \left(1-\frac{1}{\beta }\right)}$$

where β and δ are critical exponents.

**Figure 8:**
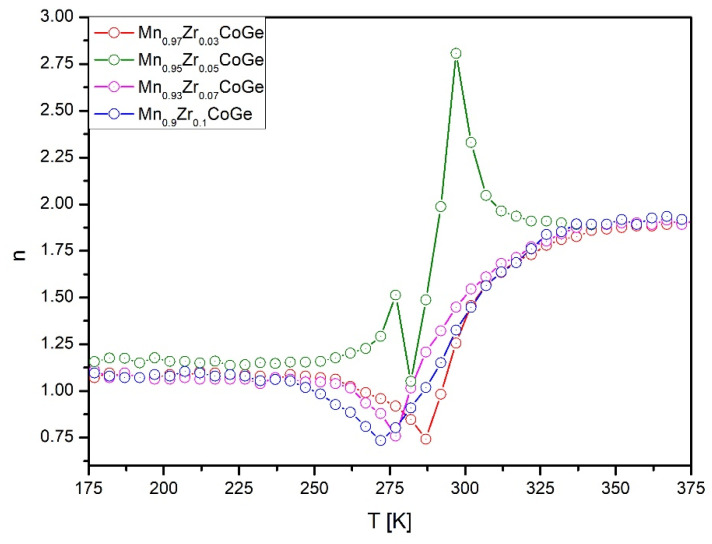
The temperature dependences of the exponent n calculated for all investigated samples.

The temperature dependences of exponent n are presented in Figure 8 for all studied samples. In the case of samples with Zr contents x = 0.03, 0.07, and 0.1, the n vs. T curves are typical for the second order phase transition. Moreover, the values revealed in the vicinity of the T_C_ amount of 0.74, 0.76, and 0.73, for Mn_0.97_Zr_0.03_CoGe, Mn_0.93_Zr_0.07_CoGe, and Mn_0.9_Zr_0.1_CoGe, respectively. These values are similar, which suggests that values of critical exponents are close to others. However, the n vs. T curve constructed for Mn_0.95_Zr_0.05_CoGe is typical for samples, which manifest a first order phase transition and structural transformation [31,35]. An occurrence of a structural peak in the vicinity of 275 K corresponds very well to the peak in the differential scanning calorimetry (DSC) curve presented in [22].

## 4. Conclusions

In this paper, we investigated the effect of the partial substitution of Mn by Zr in the MnCoGe alloys on the structure and magnetic properties. The coexistence of the orthorhombic TiNiSi-type phase and hexagonal Ni_2_In- type phases was found for all investigated samples. Moreover, the XRD studies supported by the Rietveld analysis allowed us to detect the structural transformation. We found a gradual decrease in the Curie temperature with the increase of the Zr content in the alloy composition. For the sample with Zr content x = 0.05, a significant increase of the magnetic entropy change was achieved, induced by a magnetostructural phase transition. In the case of other samples, the gradual decrease of the ΔS_M_ was calculated. The analysis of the temperature dependence of exponent n ($\Delta S_{M}=C\cdot {\left(B_{\mathrm{MAX}}\right)}^{n}$) proved an occurrence of a magnetostructural transition in the Mn_0.95_Zr_0.05_CoGe alloy sample.

## Figures and Tables

**Table 1:** The results of the Rietveld analysis for all investigated samples.

Alloy	Crystalline Phase	Lattice Parameter [Å] ± 0.001	Volume Fraction [%]
Mn_0.97_Zr_0.03_CoGe	hex Ni_2_In- type	a = 4.072	93
		c = 5.282	
	ort NiTiSi- type	a = 5.939	7
		b = 3.825	
		c = 7.052	
Mn_0.95_Zr_0.05_CoGe	hex Ni_2_In- type	a = 4.073	92
		c = 5.283	
	ort NiTiSi- type	a = 5.940	8
		b = 3.825	
		c = 7.053	
Mn_0.93_Zr_0.0.07_CoGe	hex Ni_2_In- type	a = 4.079	82
		c = 5.284	
	ort NiTiSi- type	a = 5.940	18
		b = 3.827	
		c = 7.054	
Mn_0.9_Zr_0.1_CoGe	hex Ni_2_In- type	a = 4.081	72
		c = 5.285	
	ort NiTiSi- type	a = 5.941	28
		b = 3.827	
		c = 7.055	

**Table 2:** The magnetic entropy change ΔS_M_ and refrigerant capacity RC for the Mn_0.97_Zr_0.03_CoGe, Mn_0.95_Zr_0.05_CoGe, Mn_0.93_Zr_0.07_CoGe, and Mn_0.9_Zr_0.1_CoGe alloys.

Alloy	Δ(μ_0_H) [T]	ΔS_M_ [J (kg K)^−1^]	RC [J kg^−1^]
Mn_0.97_Zr_0.03_CoGe	1	1.38	29
	2	3.18	67
	3	4.41	92
	4	5.51	139
	5	6.93	195
Mn_0.95_Zr_0.05_CoGe	1	2.64	37
	2	6.34	99
	3	8.71	174
	4	12.02	296
	5	13.42	425
Mn_0.93_Zr_0.07_CoGe	1	1.13	41
	2	1.73	71
	3	2.46	114
	4	3.33	165
	5	3.96	246
Mn_0.9_Zr_0.1_CoGe	1	0.66	33
	2	1.35	78
	3	1.97	121
	4	2.42	177
	5	2.94	219

## Data Availability

The data presented in this study are available on request from the corresponding author.
